# Supplementary material for: Splice-Junction-Based Mapping of Alternative Isoforms in the Human Proteome
Source: Cell Rep. Author manuscript; Available in PMC 2020 Jan 15. (PMC6961840; doi:10.1016/j.celrep.2019.11.026)
Supplement: 3 [file NIHMS1546469-supplement-3.zip › DF2/PXD009737/TestisLysC-161-P51531-KDSSGRQLSEVFIQLPSRK.pdf]

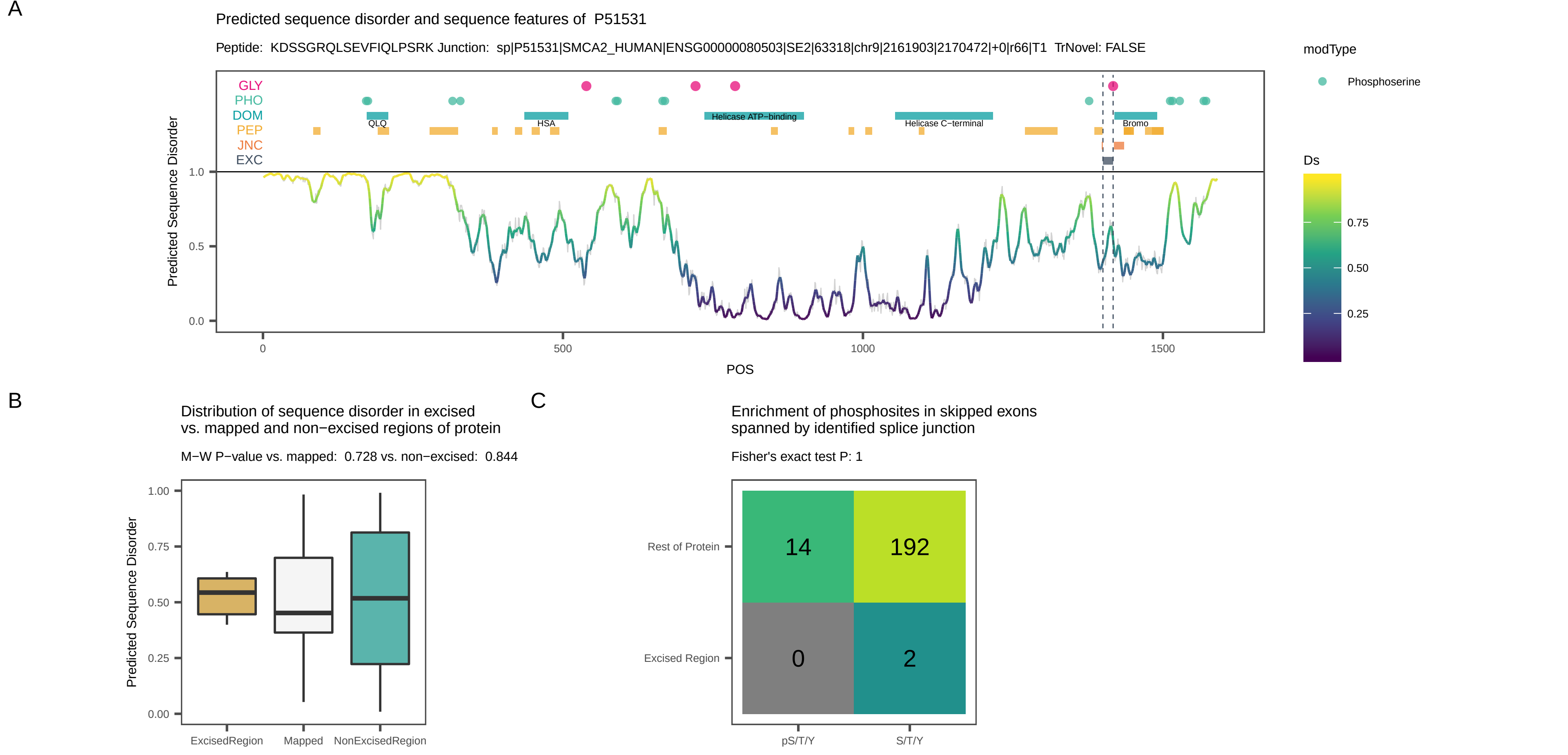
A
Predicted sequence disorder and sequence features of P51531
Peptide: KDSSGRQLSEVFIQLPSRK Junction: sp|P51531|SMCA2_HUMAN|ENSG00000080503|SE2|63318|chr9|2161903|2170472|+0|r66|T1 TrNovel: FALSE
GLY
PHO
DOM
PEP
JNC
EXC
QLQ
HSA
Helicase ATP−binding
Helicase C−terminal
Bromo
Predicted Sequence Disorder
1.0
0.5
0.0
0
500
1000
1500
POS
modType
Phosphoserine
Ds
0.75
0.50
0.25
B
Distribution of sequence disorder in excised
vs. mapped and non−excised regions of protein
M−W P−value vs. mapped: 0.728 vs. non−excised: 0.844
Predicted Sequence Disorder
1.00
0.75
0.50
0.25
0.00
ExcisedRegion
Mapped
NonExcisedRegion
C
Enrichment of phosphosites in skipped exons
spanned by identified splice junction
Fisher's exact test P: 1
Rest of Protein
Excised Region
14
192
0
2
pS/T/Y
S/T/Y
